# Clutch Destruction by Common Cuckoos ( *Cuculus canorus* ) During the Laying Stage of Vinous‐Throated Parrotbills ( *Sinosuthora webbiana* ): First Video Evidence

**DOI:** 10.1002/ece3.73387

**Published:** 2026-04-17

**Authors:** Yunkyoung Lee, Wonseok Jang

**Affiliations:** ^1^ Department of Biology Kyunghee University Seoul Republic of Korea; ^2^ National Institute of Ecology Seocheon Republic of Korea; ^3^ Department of Biological Sciences Seoul National University Seoul Republic of Korea

**Keywords:** brood parasitism, clutch destruction, *Cuculus canorus*, egg recognition, host–parasite coevolution, *Sinosuthora webbiana*

## Abstract

Brood parasitism by the Common Cuckoo (*Cuculus canorus*) depends on precise timing, with females typically laying during the host's laying phase to ensure their chick hatches first. While cuckoos are known to remove one or two host eggs during laying, complete destruction of host clutches at this stage has been rarely documented. Here, we present five video‐documented cases of cuckoo clutch destruction during laying in Vinous‐throated Parrotbills (*Sinosuthora webbiana*), a semi‐colonial host that breeds synchronously. Across visits lasting only 2–72 s, cuckoos swallowed, broke, or carried away host eggs, resulting in the loss of 1–4 eggs per event and eventual desertion of all nests. Such behavior at the very stage most favorable for parasitism appears paradoxical, yet may serve conditional functions: resetting host reproduction when cuckoos are not ready to lay, or eliminating nests deemed unsuitable under synchronous breeding conditions where many options are available simultaneously. A further consequence is that hosts lose opportunities to reinforce egg‐recognition templates, potentially weakening defenses in subsequent attempts. These findings highlight the importance of early‐stage interactions in the host–parasite arms race and suggest that laying‐stage clutch destruction represents a previously overlooked element of cuckoo reproductive behavior.

## Graphical abstract

We present the first video‐documented evidence of clutch destruction by Common Cuckoos during the laying stage of Vinous‐throated Parrotbills. This behavior, observed across five cases, led to complete clutch loss and host desertion. Our findings expand the known timeline of brood parasite aggression and offer new insights into host–parasite coevolution.

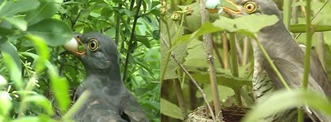

## 1 Introduction

Coevolution between avian brood parasites and their hosts unfolds across successive stages of the breeding cycle, with hosts evolving defenses and parasites counter‐adapting to overcome them (Davies 2011). At the earliest stage, hosts attempt to reduce the risk of parasitism altogether by selecting concealed nest sites (Moskát and Honza 2000), nesting in colonies (Medina and Langmore 2019), or remaining near the nest while actively responding to intruders (e.g., alarm calling or mobbing) (Wang and Yang 2020) whereas parasites counter these defenses by monitoring host activity, timing their visits (Davies 2000), and laying rapidly when opportunities arise (Andou et al. 2005).

Much research on Common Cuckoos has focused on the egg stage, particularly on host egg recognition and rejection and the corresponding evolution of egg mimicry and crypsis in the parasite (Brooke and Davies 1988; Moksnes et al. 1991; Stokke et al. 2017). By contrast, interactions occurring before or during the early laying period have received less attention, particularly behaviors such as clutch destruction that occur in the absence of immediate parasitism.

Brood parasitism by the Common Cuckoo ( *Cuculus canorus* ) depends on precise timing, as females must synchronize laying with that of the host to ensure their chick hatches ahead of or alongside host young (Davies 2000). Typically, cuckoos exploit host nests during the laying phase by depositing a single egg within seconds and removing one or more host eggs, a behavior that appears to be an integral component of cuckoo parasitism (Jelínek et al. 2014), although laying may occasionally occur in empty nests (Honza et al. 2022).

Adult brood parasites have also been documented killing host nestlings (Kim and Yamagishi 1999; Šulc et al. 2020; Zhang et al. 2024), although the adaptive significance of this rare behavior remains uncertain. A comprehensive review by Šulc et al. (2020) compiled 43 such events across five species, including the Common Cuckoo and Brown‐headed Cowbird ( *Molothrus ater* ). These attacks have been interpreted mainly as retaliation against host rejection (the “Mafia” hypothesis) (Soler et al. 1995; Šulc et al. 2020; Zahavi 1979) or as attempts to induce host re‐laying to create new parasitic opportunities, a mechanism often referred to as the ‘farming hypothesis’ (Hoover and Robinson 2007).

Egg predation by cuckoos has also been reported, although such events appear to be rare. For example, Wyllie (1975) documented nine cases of complete egg predation in Reed Warbler nests, and Gehringer (1979) described three additional cases. Building on these observations, we report five video‐documented cases of clutch predation by Common Cuckoos during the early laying stage of Vinous‐throated Parrotbills, three involving complete clutch destruction and two involving partial clutch predation. These events expand the known behavioral repertoire of cuckoos and indicate that destructive behaviors may occur earlier in the breeding cycle than previously recognized.

Our study provides the first detailed video documentation of egg destruction during the host laying stage and examines its potential adaptive significance within the temporal dynamics of brood parasitism.

## 2 Methods

Fieldwork was conducted at Puyong‐ri, Yangseo‐myeon, Yangpyeong‐gun, Gyeonggi‐do, South Korea (37°32′N, 127°20′E) between early April and late July 2007. The study area consists of low hills (~200 m) with mixed forest, farmland bordered by hedgerows, cultivated fields, and a river 30–40 m wide with reed beds (see Lee et al. 2009 for further details).

The Vinous‐throated Parrotbill ( *Sinosuthora webbiana* ) is a small passerine (~11 g) that builds open‐cup nests and is occasionally parasitized by the Common Cuckoo ( *Cuculus canorus* ) in Korea (Kim et al. 1995). This species exhibits a semi‐colonial breeding system, with nest dispersion ranging from solitary to loosely clustered (Kim et al. 1995; Lee et al. 2009). In our study area, breeding occurred in local aggregations of multiple simultaneously active nests, typically within a 200 m radius (mean colony size = 2.8 nests, range = 0–8, *n* = 90, sampled in 2007), with substantial temporal overlap in egg laying. Both males and females share incubation duties and exhibit egg recognition, rejecting non‐mimetic cuckoo eggs (Lee et al. 2005).

Parrotbill clutches contain either white or turquoise‐blue eggs (sometime paler blue) without spots, with blue eggs being more common in the study population (approximately 65% blue and 35% white) and cuckoo eggs found in this population closely match the blue type, or pale blue; white cuckoo eggs have not been reported from this population or elsewhere in parrotbill hosts to date (Kim et al. 1995). Previous observations have also documented destructive cuckoo behavior during the chick‐rearing phase, including nestling killing in this host species (Kim and Yamagishi 1999).

Because Vinous‐throated Parrotbills are typically double‐brooded, with the first laying peak occurring before the arrival of Common Cuckoos in the study area, only nests active after cuckoo arrival (approximately mid‐May in the study area) are available for parasitism. Based on this definition, the parasitism rate in the study population in 2007 was approximately 11% (5 of 45 nests). These host–parasite interactions indicate frequent and close contact between parrotbills and cuckoos throughout the breeding cycle, providing opportunities to observe rare cases of destructive behavior of the parasite.

To investigate host parental behavior, we video‐monitored 24 parrotbill nests during egg deposition. Digital video cameras (JVC GZ‐MG70KR and Sony Handycam SR62) were typically deployed from the late morning and recorded continuously for 4–9 h (mean recording duration: 6.5 h), depending on battery capacity and weather conditions. The video recordings were originally collected to document parental identity (via color rings) and visit frequency during the host laying period; however, the continuous monitoring protocol remains appropriate for the present study as it allowed direct observation of cuckoo behavior during host egg laying. Cameras were mounted on tripods, positioned 3–12 m from nests, and concealed with camouflage covers painted with acrylic and supplemented with natural vegetation to minimize disturbance.

We obtained video recordings from five host nests in which clutch‐destruction events by cuckoos were observed. In two recordings, the cuckoo could be confidently identified as a female based on visible plumage characteristics, most notably a rufous‐tinged upper breast, together with weak barring on the breast and neck and an overall gray appearance consistent with female Common Cuckoos (Payne et al. 2020; Noh et al. 2016). In the remaining three cases, reliable sex identification was not possible due to vegetation cover, viewing angle, or limited image quality. Host nest searching and inspection during the laying period have been reported to be primarily associated with female Common Cuckoos, which require detailed information on nest location and timing for successful parasitism (Davies 2000; Nakamura et al. 2005).

## 3 Results

Video recordings captured five cases of clutch destruction by Common Cuckoos during the laying stage of Vinous‐throated Parrotbills (Table 1; Videos [Link], [Link], [Link], [Link], [Link]). All nests had been monitored since construction, and in every case the host deserted following destruction, with no further eggs laid.

**TABLE 1:** Summary of five video‐documented cases of clutch destruction by Common Cuckoos during host laying in Vinous‐throated Parrotbills. In all cases, the host deserted the nest following destruction; no further eggs were laid. Each case corresponds to a distinct and independent host nest. All nests were located during the nest‐building phase. In most nests, video recordings were initiated from the day the first egg was laid and were repeated during the egg‐laying period.

Video	Date	Host laying day	Cuckoo visit time	Duration (s)	No. of eggs destroyed	Host egg color	Notes
S1	4 Jun 2007	Day 3	16:23	66	2/3	White	One egg swallowed, one carried; clutch deserted
S2	4 Jun 2007	Day 2	15:55	74	2/2	Blue	Two eggs broken and contents consumed
S3	15 Jun 2007	Day 4	18:21	70	4/4	Blue	Two eggs swallowed, one broken and consumed, last egg carried
S4	16 Jun 2007	Day 1	18:52	12	1/1	White	Single egg carried away
S5	16 Jun 2007	Day 5	18:59	2	1/5	White	Flushed by the host; one egg seized, the rest of the eggs absent by next day

**FIGURE 1:**
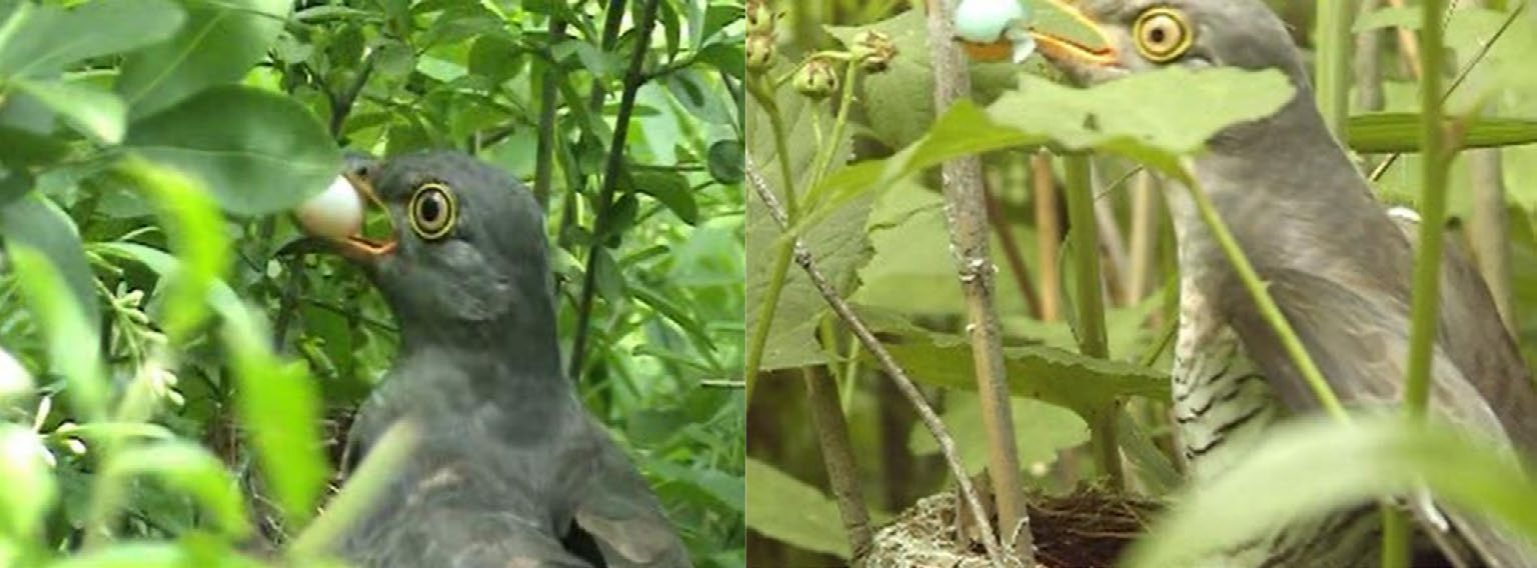
Video‐documented case of clutch destruction by a female Common Cuckoo (*Cuculus canorus*) during the laying phase of a Vinous‐throated Parrotbill (*Sinosuthora webbiana*). (Left): The cuckoo swallowed one white host egg, then carried away another after a brief attempt to break it (see Video S1). (Right): The cuckoo broke both turquoise‐blue eggs, fully consumed the first and only partially the second before discarding it (see Video S2).

Events occurred between 4 and 16 June 2007, spanning laying Days 1–5. Cuckoo visits took place in the late afternoon to evening (15:55–18:59) and lasted 2–72 s. Destruction involved swallowing eggs whole, breaking eggs and consuming the contents, or carrying eggs away in the bill (Figure 1). Clutch loss ranged from partial (Video S1, two of three eggs removed) to complete predation (Videos [Link], [Link], [Link]), while in Video S5 a single egg was taken, and the remaining clutch had disappeared by the following day.

## 4 Discussion

Our observations provide the first video‐documented evidence that Common Cuckoos may destroy host clutches during the laying stage, a period usually regarded as the prime opportunity for successful parasitism. Importantly, this behavior involved consumption of host eggs and therefore constitutes egg predation, which is distinct from nestling ejection reported at later breeding stages. Previous reports of destructive behavior in cuckoos have primarily described nestling removal during the chick‐rearing period, a behavior interpreted as deliberate interference with host reproduction once parasitism is no longer possible (Šulc et al. 2020). By contrast, our records show that cuckoos can also eliminate host clutches before incubation begins, extending the known range of destructive behavior into an earlier phase of reproduction.

That such events occur during the laying phase appears paradoxical, since this is normally the most favorable stage for cuckoos to lay and synchronize their chick's hatching with the host. Although egg consumption alone could represent simple predation, the timing and context of these events suggest that clutch destruction may occur as part of a conditional response rather than merely opportunistic feeding. Female cuckoos are known to inspect host nests even when an egg is not yet ready to be laid, presumably to assess nest suitability and timing for future parasitism (Davies 2000); clutch destruction during such inspections may therefore reflect decision‐making under these constraints.

Two non‐exclusive hypotheses may account for this behavior. First, strategic nest elimination may occur if the cuckoo is not physiologically ready to lay: females produce eggs at 2–3 day intervals (Nakamura et al. 2005) and may sometimes encounter suitable nests before a mature egg is available. In such cases, destruction could reset host reproduction and bring re‐nesting into synchrony with the cuckoo's own cycle, extending the interference hypothesis previously applied to nestling‐stage predation.

Second, flexible nest selection may play a role. Female cuckoos are known to inspect host nests prior to laying, presumably to monitor nest availability and timing for future parasitism (Moksnes et al. 2000; Nakamura et al. 2005). In species that breed semi‐colonially, such as the Vinous‐throated Parrotbill, multiple nests are clustered in space and often synchronized in timing (Kim et al. 1995; Lee et al. 2009). Under these conditions, cuckoos may encounter several potential nests within a short time window. When several nests are available simultaneously, cuckoos may be forced to select only a subset of nests for parasitism, and clutch destruction may function as a means of reducing immediate options and shifting host re‐nesting into a later period that better matches the cuckoo's own laying schedule. Thus, the semi‐colonial and synchronous breeding of parrotbills may increase the likelihood of clutch destruction as part of a conditional decision‐making strategy. A broader comparative approach would help to clarify the generality of this pattern. Comparing egg predation behavior across host species that differ in breeding synchrony and spatial nesting structure may reveal whether clutch destruction during laying is more likely to occur in hosts with highly synchronized or semi‐colonial breeding systems.

Compared with destruction during the nestling stage, clutch destruction during the laying period occurs when host investment is still minimal. Although complete nestling predation can also result in desertion, destruction at the laying stage is more likely to trigger rapid and predictable re‐nesting, thereby maintaining temporal synchrony with the cuckoo's own reproductive schedule.

An additional consequence of destruction at this stage is that hosts lose opportunities to reinforce egg‐recognition templates, which typically develop between host egg‐laying and incubation (Rothstein 1975; Lotem et al. 1995; Hauber et al. 2006). This disruption may weaken host defenses and increase the likelihood of parasitic egg acceptance in subsequent nesting attempts, even if mimicry is imperfect.

That all recorded events were brief and occurred in the late afternoon, coinciding with normal cuckoo laying times, suggests that clutch destruction is integrated into the same behavioral repertoire as parasitism. Although rarely documented, such events may be under‐detected rather than exceptional, given their unpredictability and the difficulty of continuous observation.

These findings highlight the importance of early‐stage interactions in the host–parasite arms race and indicate that clutch destruction during laying represents a previously overlooked element of cuckoo reproductive behavior.

## Author Contributions

**Yunkyoung Lee:** conceptualization (equal), data curation (equal), formal analysis (equal), funding acquisition (lead), investigation (equal), methodology (equal), resources (equal), writing – original draft (lead), writing – review and editing (equal). **Wonseok Jang:** conceptualization (equal), data curation (equal), investigation (equal), methodology (equal), resources (equal), supervision (lead), visualization (equal), writing – review and editing (equal).

## Funding

This work was supported by the National Institute of Ecology, grant NIE‐B‐2025‐13, NIE‐A‐2025‐01.

## Conflicts of Interest

The authors declare no conflicts of interest.

## Supporting information


**Video S1:** A female Common Cuckoo removes eggs from a Vinous‐throated Parrotbill nest on laying Day 3 (4 June 2007, 16:23 h; 66 s). One egg (white) was swallowed and another carried away, resulting in clutch desertion.

**Video S2:** A female Common Cuckoo visits a Vinous‐throated Parrotbill nest on laying Day 2 (4 June 2007, 15:55 h; 74 s). Both host eggs (blue) were broken and their contents consumed.

**Video S3:** A Common Cuckoo visits a Vinous‐throated Parrotbill nest on laying Day 4 (15 June 2007, 18:21 h; 70 s). All four host eggs (blue) were destroyed: two swallowed, one broken and consumed, and one carried away.

**Video S4:** A Common Cuckoo visits a Vinous‐throated Parrotbill nest on laying Day 1 (16 June 2007, 18:52 h; 12 s) and carries away the single host egg (white).

**Video S5:** A Common Cuckoo visits a Vinous‐throated Parrotbill nest on laying Day 5 (16 June 2007, 18:59 h; 2 s). The host was flushed, and one egg (white) was seized; all remaining eggs were absent by the following day.

## Data Availability

All data supporting this study are provided in the five video files submitted as Supporting Information.
